# Benzyl 3-[(*E*)-(furan-2-yl)methyl­idene]-2-methyldithio­carbazate

**DOI:** 10.1107/S1600536812035520

**Published:** 2012-08-23

**Authors:** Benu K. Dey, Sebastian Suarez, Biplab Ganguly, Fabio Doctorovich, Tapashi G. Roy

**Affiliations:** a University of Chittagong, Chittagong 4331, Bangladesh; b Departamento de Química Inorgánica Analítica y Química Física, INQUIMAE-CONICET, Facultad de Ciencias Exactas y Naturales, Universidad de Buenos Aires, Argentina

## Abstract

In the title compound, C_14_H_14_N_2_OS_2_, the furan ring exhibits rotational disorder over two orientations, with an occupancy ratio of 0.508 (7):0.492 (7). The furan and phenyl rings form dihedral angles of 8.2 (6) (major occupancy component), 14.8 (6) (minor occupancy component) and 73.65 (9)°, respectively, with the central residue (C_4_N_2_S_2_), indicating a twisted conformation for the mol­ecule. The methyl group and the thione S atom are *syn* and the conformation about the imine bond is *E*. In the crystal, C—H⋯π inter­actions involving the phenyl ring are observed.

## Related literature

For background to the biological activity of S-containing ligands, see: Hazari *et al.* (2012 ▶). For related structures, see: Shan *et al.* (2008 ▶); Ganguly *et al.* (2011 ▶). For a similar compound with a thio­phene instead of a furan ring, see: Hazari *et al.* (2012 ▶).
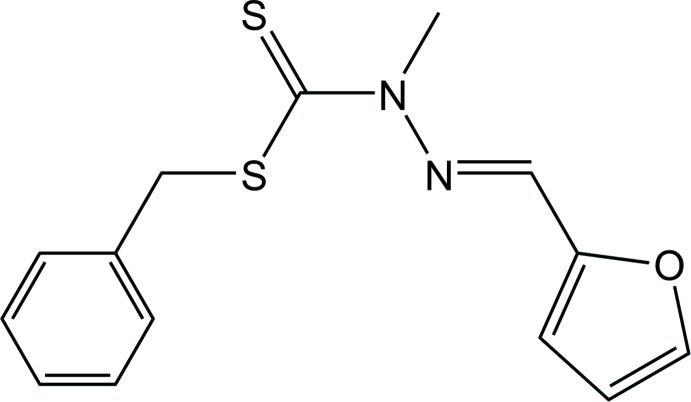


## Experimental

### 

#### Crystal data

C_14_H_14_N_2_OS_2_
*M* *_r_* = 290.39
Monoclinic, 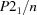

*a* = 6.0415 (3) Å
*b* = 20.4840 (11) Å
*c* = 11.8959 (7) Å
β = 101.601 (5)°
*V* = 1442.09 (14) Å^3^
*Z* = 4
Mo *K*α radiation
μ = 0.36 mm^−1^
*T* = 298 K
0.5 × 0.5 × 0.3 mm

#### Data collection

Oxford Diffraction Gemini CCD S Ultra diffractometer
Absorption correction: multi-scan (*CrysAlis PRO*; Oxford Diffraction, 2009 ▶) *T*_min_ = 0.850, *T*_max_ = 0.897
21725 measured reflections
3361 independent reflections
2393 reflections with *I* > 2σ(*I*)
*R*_int_ = 0.033

#### Refinement

*R*[*F* ^2^ > 2σ(*F* ^2^)] = 0.040
*wR*(*F* ^2^) = 0.090
*S* = 1.06
3361 reflections
219 parameters
12 restraints
H-atom parameters constrained
Δρ_max_ = 0.16 e Å^−3^
Δρ_min_ = −0.17 e Å^−3^

### 

Data collection: *CrysAlis PRO* (Oxford Diffraction, 2009 ▶); cell refinement: *CrysAlis PRO*; data reduction: *CrysAlis PRO*; program(s) used to solve structure: *SHELXS97* (Sheldrick, 2008 ▶); program(s) used to refine structure: *SHELXL97* (Sheldrick, 2008 ▶); molecular graphics: *ORTEP-3 for Windows* (Farrugia, 1997 ▶); software used to prepare material for publication: *WinGX* (Farrugia, 1999 ▶).

## Supplementary Material

Crystal structure: contains datablock(s) I, global. DOI: 10.1107/S1600536812035520/bh2449sup1.cif

Structure factors: contains datablock(s) I. DOI: 10.1107/S1600536812035520/bh2449Isup2.hkl

Supplementary material file. DOI: 10.1107/S1600536812035520/bh2449Isup3.cml

Additional supplementary materials:  crystallographic information; 3D view; checkCIF report

## Figures and Tables

**Table 1:** Hydrogen-bond geometry (Å, °) Table 1 ▶. *Cg* is the centroid of the phenyl ring

*D*—H⋯*A*	*D*—H	H⋯*A*	*D*⋯*A*	*D*—H⋯*A*
C7—H7*B*⋯*Cg* ^i^	0.97	2.88	3.560 (2)	128
C13—H13*A*⋯*Cg* ^ii^	0.93	2.80	3.62 (2)	149

Symmetry codes: (i) 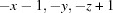; (ii) 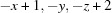.

**Fig. 1.**
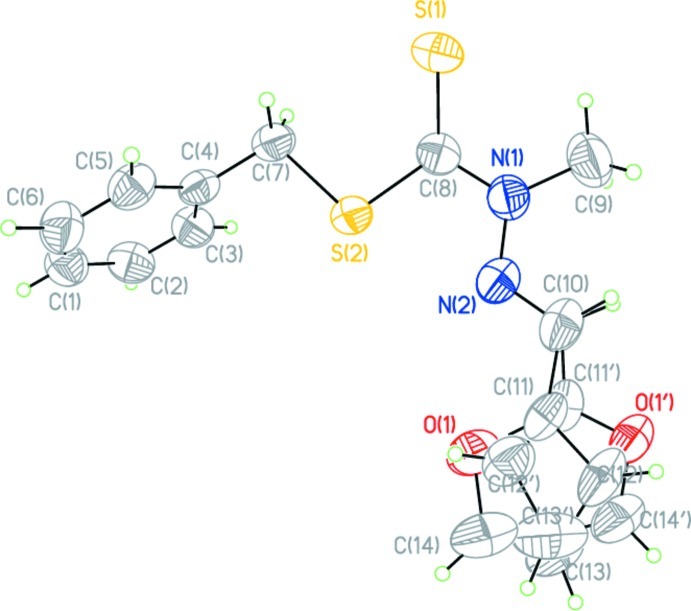
The molecular structure of the title molecule, showing displacement ellipsoids at the 50% probability level for non-H atoms.

**Fig. 2.**
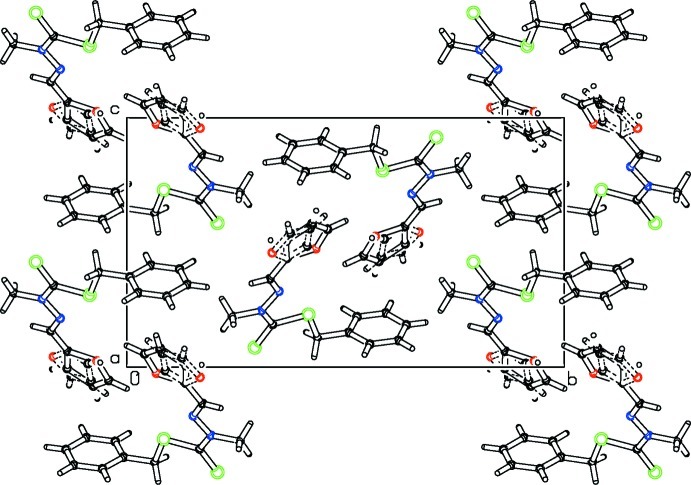
Crystal packing for the title compound viewed along a.

## supplementary crystallographic information

### Comment

As a continuation of systematic studies into the synthesis, characterization and biological activities of substituted Schiff base ligands and their metal complexes (Ganguly *et al.*, 2011; Hazari *et al.*, 2012), the present investigation is an attempt to prepare complexes of vanadium(IV) and molybdenum(VI) with the title Schiff base ligand, benzyl 2-methyl-3-[(*E*)-(furan-2-yl)-methylidene]dithiocarbazate. Crystals of the title compound were isolated (see *Experimental*) and characterized crystallographically.

In the title compound (Fig. 1), C_14_H_14_N_2_OS_2_, the furan ring exhibits rotational disorder over two orientations, with an occupancy of 0.5 for each orientation. The thione S atom and methyl group are *syn* and the conformation about the imine N2═C10 bond [1.281 (2) Å] is *E*, in agreement with similar structures (Hazari *et al.*, 2012).

The eight atoms of the central residue (S1, S2, N1, N2, C7, C8, C9 and C10) are co-planar having a r.m.s. deviation for the fitted atoms of 0.002 Å. The maximum deviations from this plane are 0.043 (2) Å for the N2 atom and -0.033 (3) Å for the N1 atom. The molecule is twisted, the dihedral angles between the C_4_N_2_S_2_ residue and the pendent 2-furanyl and phenyl rings being 14.8 (6) [or 8.22 (6) for the disordered part of the furanyl] and 73.65 (9)° respectively, as found in a similar compound (Shan *et al.*, 2008).

In the crystal, molecules assemble into a three-dimensional architecture by π ··· π stacking between 2-furanyl rings [Cg_1_···Cg_1_^iii^ = 4.467 (7) Å, Cg_1_ is the centroid of ring O1, C11, C12, C13, C14; symmetry code: iii 4-*x*, -*y*, 2-*z*], and C–H ··· π interactions, involving the phenyl ring as acceptor (see Table 1 and Fig. 2).

### Experimental

Single crystals of the title compound were prepared by following three steps.

Step 1 (Hazari *et al.*, 2012). Synthesis of *N*-methyl-*S*-benzyldithiocarbazate. Potassium hydroxide (11.5 g) was dissolved in 60 ml of 90% ethanol and the mixture was cooled down to 273 K in an ice bath. Methyl hydrazine (11.1 ml) was added slowly with mechanical stirring. A solution of CS_2_ (12 ml) was added dropwise from a burette with constant stirring over a period of 1 h. During the addition of CS_2_, the temperature of the reaction mixture was not allowed to rise above 279 K. A yellow colour was obtained. After adding carbon disulfide, benzyl chloride (25 ml) was added from a burette dropwise with vigorous mechanical stirring. After complete addition, the mixture was stirred for further 15 min, whereupon shining crystals appeared. The product was separated by filtration, washed with water, recrystallized from ethanol and dried in a vacuum desiccator over silica gel. Yield: 14.20 g. m.p. 373–374 K.

Step 2. Synthesis of the title molecule. A hot solution of furan-2-carbaldehyde (10 mmol) in absolute ethanol (40 ml) was mixed with a hot solution of *N*-methyl-*S*-benzyldithiocarbazate (10 mmol) in 40 ml of the same solvent. The mixture was refluxed for 6 h on a water bath. After reducing the volume, an off white product appeared which was filtered off. This product was washed with ethanol several times and dried in a vacuum desiccator over silica gel. Yield: 1.65 g. m.p. 432–434 K.

Step 3. Crystallization. The product was dissolved in ethanol to which half volume of petroleum ether was added (2:1 *v*/*v*, 10 ml ethanol and 5 ml petroleum ether). The solution was left for several days after which crystals of the title compound deposited.

### Refinement

All H atoms were placed in idealized positions and allowed to ride on their parent C atoms, with C—H bond lengths fixed to 0.93 (aromatic CH), 0.97 (methylene CH_2_) or 0.96 Å (methyl CH_3_). Displacement parameters were taken as *U*_iso_(H) = 1.5*U*_eq_(C9) for the methyl group and *U*_iso_(H) = 1.2*U*_eq_(carrier C) otherwise. The furan ring exhibits rotational disorder over two orientations. The occupancies for all sites were fixed to 0.5, since the refined occupancy for each part was very close to that distribution. In order to approximate the expected geometry for both furan groups, their bond lengths were restrained to be identical, with an effective standard deviation of 0.01 Å (command *SAME* in *SHELXL97*; Sheldrick, 2008).

### Crystal data

**Table:**

C_14_H_14_N_2_OS_2_	*F*(000) = 608
*M**_r_* = 290.39	*D*_x_ = 1.338 Mg m^−^^3^
Monoclinic, *P*2_1_/*n*	Melting point: 432 K
Hall symbol: -P 2yn	Mo *K*α radiation, λ = 0.71073 Å
*a* = 6.0415 (3) Å	Cell parameters from 4212 reflections
*b* = 20.4840 (11) Å	θ = 4.0–28.9°
*c* = 11.8959 (7) Å	µ = 0.36 mm^−^^1^
β = 101.601 (5)°	*T* = 298 K
*V* = 1442.09 (14) Å^3^	Prism, green
*Z* = 4	0.5 × 0.5 × 0.3 mm

### Data collection

**Table:**

Oxford Diffraction Gemini CCD S Ultra diffractometer	3361 independent reflections
Graphite monochromator	2393 reflections with *I* > 2σ(*I*)
Detector resolution: 16.1158 pixels mm^-1^	*R*_int_ = 0.033
ω scans	θ_max_ = 27.9°, θ_min_ = 4.0°
Absorption correction: multi-scan (*CrysAlis PRO*; Oxford Diffraction, 2009)	*h* = −7→7
*T*_min_ = 0.850, *T*_max_ = 0.897	*k* = 0→26
21725 measured reflections	*l* = 0→15

### Refinement

**Table:**

Refinement on *F*^2^	Primary atom site location: structure-invariant direct methods
Least-squares matrix: full	Secondary atom site location: difference Fourier map
*R*[*F*^2^ > 2σ(*F*^2^)] = 0.040	Hydrogen site location: inferred from neighbouring sites
*wR*(*F*^2^) = 0.090	H-atom parameters constrained
*S* = 1.06	*w* = 1/[σ^2^(*F*_o_^2^) + (0.0275*P*)^2^ + 0.3918*P*] where *P* = (*F*_o_^2^ + 2*F*_c_^2^)/3
3361 reflections	(Δ/σ)_max_ < 0.001
219 parameters	Δρ_max_ = 0.16 e Å^−^^3^
12 restraints	Δρ_min_ = −0.17 e Å^−^^3^
0 constraints	

### Fractional atomic coordinates and isotropic or equivalent isotropic displacement parameters (Å^2^)

**Table:**

	*x*	*y*	*z*	*U*_iso_*/*U*_eq_	Occ. (<1)
S1	0.84126 (9)	0.20754 (3)	0.57831 (5)	0.06926 (18)	
S2	0.99621 (8)	0.08488 (2)	0.71050 (4)	0.05239 (14)	
N1	1.2300 (3)	0.19263 (7)	0.72613 (14)	0.0561 (4)	
N2	1.3649 (2)	0.15222 (8)	0.80423 (13)	0.0554 (4)	
C1	0.5997 (3)	−0.13887 (10)	0.66897 (17)	0.0637 (5)	
H1A	0.5707	−0.1828	0.6791	0.076*	
C2	0.7562 (4)	−0.12057 (10)	0.60675 (18)	0.0656 (5)	
H2A	0.8344	−0.1522	0.5744	0.079*	
C3	0.7987 (3)	−0.05537 (10)	0.59175 (17)	0.0605 (5)	
H3A	0.9062	−0.0436	0.5494	0.073*	
C4	0.6844 (3)	−0.00718 (9)	0.63849 (14)	0.0471 (4)	
C5	0.5281 (3)	−0.02642 (10)	0.70175 (16)	0.0580 (5)	
H5A	0.4504	0.005	0.735	0.07*	
C6	0.4856 (3)	−0.09170 (11)	0.71639 (19)	0.0676 (6)	
H6A	0.3786	−0.1039	0.7588	0.081*	
C7	0.7263 (3)	0.06390 (9)	0.61993 (16)	0.0554 (5)	
H7A	0.6067	0.0902	0.6403	0.066*	
H7B	0.7302	0.0719	0.54	0.066*	
C8	1.0304 (3)	0.16650 (9)	0.67204 (15)	0.0496 (4)	
C9	1.3012 (4)	0.25833 (10)	0.7034 (2)	0.0813 (7)	
H9A	1.1825	0.2797	0.6503	0.122*	
H9B	1.4343	0.2561	0.671	0.122*	
H9C	1.3339	0.2826	0.7738	0.122*	
C10	1.5598 (3)	0.17332 (11)	0.85386 (18)	0.0663 (6)	
H10A	1.6166	0.2134	0.8364	0.08*	0.508 (7)
H10B	1.5948	0.2156	0.8347	0.08*	0.492 (7)
O1	1.6236 (10)	0.0685 (3)	0.9740 (6)	0.0728 (16)	0.508 (7)
C11	1.691 (2)	0.1271 (8)	0.9441 (14)	0.055 (3)	0.508 (7)
C12	1.9117 (12)	0.1360 (5)	1.0053 (7)	0.081 (2)	0.508 (7)
H12A	2.0061	0.1716	1.003	0.097*	0.508 (7)
C13	1.960 (4)	0.0774 (10)	1.0732 (16)	0.083 (4)	0.508 (7)
H13A	2.095	0.0684	1.124	0.1*	0.508 (7)
C14	1.7950 (12)	0.0413 (4)	1.0535 (6)	0.088 (2)	0.508 (7)
H14A	1.7869	0.0008	1.0878	0.106*	0.508 (7)
O1'	1.9236 (7)	0.1675 (2)	0.9587 (4)	0.0668 (14)	0.492 (7)
C11'	1.720 (2)	0.1418 (9)	0.9281 (16)	0.054 (3)	0.492 (7)
C12'	1.6978 (18)	0.0874 (6)	0.9882 (9)	0.075 (2)	0.492 (7)
H12B	1.5713	0.0611	0.9855	0.09*	0.492 (7)
C13'	1.922 (5)	0.0804 (15)	1.058 (2)	0.112 (9)	0.492 (7)
H13B	1.969	0.0467	1.1097	0.134*	0.492 (7)
C14'	2.0407 (11)	0.1267 (4)	1.0379 (6)	0.078 (2)	0.492 (7)
H14B	2.1915	0.1324	1.073	0.093*	0.492 (7)

### Atomic displacement parameters (Å^2^)

**Table:**

	*U*^11^	*U*^22^	*U*^33^	*U*^12^	*U*^13^	*U*^23^
S1	0.0677 (3)	0.0587 (3)	0.0773 (4)	0.0155 (2)	0.0048 (3)	0.0102 (3)
S2	0.0496 (3)	0.0497 (3)	0.0529 (3)	0.0008 (2)	−0.00161 (19)	0.0016 (2)
N1	0.0523 (9)	0.0497 (9)	0.0666 (10)	−0.0012 (7)	0.0124 (8)	−0.0024 (8)
N2	0.0479 (9)	0.0599 (9)	0.0573 (9)	0.0000 (7)	0.0082 (7)	−0.0103 (8)
C1	0.0673 (13)	0.0523 (12)	0.0684 (13)	−0.0010 (10)	0.0061 (10)	0.0024 (10)
C2	0.0726 (13)	0.0577 (12)	0.0690 (13)	0.0165 (10)	0.0201 (11)	−0.0040 (10)
C3	0.0589 (12)	0.0627 (13)	0.0638 (12)	0.0075 (9)	0.0218 (10)	0.0016 (10)
C4	0.0400 (9)	0.0520 (10)	0.0451 (9)	0.0031 (7)	−0.0014 (7)	−0.0034 (8)
C5	0.0487 (10)	0.0617 (12)	0.0637 (12)	0.0049 (9)	0.0116 (9)	−0.0107 (10)
C6	0.0597 (12)	0.0703 (14)	0.0767 (14)	−0.0065 (10)	0.0228 (11)	−0.0010 (11)
C7	0.0479 (10)	0.0534 (11)	0.0592 (11)	0.0034 (8)	−0.0027 (8)	0.0001 (9)
C8	0.0513 (10)	0.0489 (10)	0.0509 (10)	0.0061 (8)	0.0156 (8)	−0.0048 (8)
C9	0.0736 (15)	0.0534 (13)	0.120 (2)	−0.0069 (11)	0.0259 (14)	0.0048 (13)
C10	0.0540 (12)	0.0694 (13)	0.0745 (14)	−0.0052 (10)	0.0108 (10)	−0.0231 (11)
O1	0.064 (3)	0.081 (4)	0.066 (3)	0.006 (2)	−0.004 (2)	−0.003 (2)
C11	0.037 (3)	0.078 (10)	0.051 (6)	−0.010 (5)	0.008 (4)	−0.014 (4)
C12	0.053 (3)	0.100 (7)	0.083 (5)	−0.011 (4)	−0.002 (4)	−0.034 (4)
C13	0.070 (5)	0.102 (10)	0.068 (6)	0.018 (5)	−0.006 (4)	−0.022 (5)
C14	0.080 (5)	0.114 (6)	0.064 (3)	0.031 (4)	−0.003 (3)	−0.002 (4)
O1'	0.052 (2)	0.074 (3)	0.068 (3)	−0.0043 (19)	−0.0011 (17)	−0.0178 (19)
C11'	0.053 (5)	0.056 (5)	0.056 (5)	−0.015 (4)	0.019 (4)	−0.015 (3)
C12'	0.074 (6)	0.090 (7)	0.058 (4)	−0.007 (5)	0.007 (5)	−0.003 (4)
C13'	0.13 (2)	0.129 (14)	0.070 (7)	0.048 (13)	0.003 (9)	0.010 (9)
C14'	0.060 (3)	0.094 (5)	0.070 (4)	0.013 (3)	−0.007 (3)	−0.018 (3)

### Geometric parameters (Å, º)

**Table:**

S1—C8	1.6562 (18)	C9—H9B	0.96
S2—C8	1.7566 (19)	C9—H9C	0.96
S2—C7	1.8161 (18)	C10—C11	1.529 (10)
N1—C8	1.357 (2)	C10—C11'	1.338 (11)
N1—N2	1.381 (2)	C10—H10A	0.93
N1—C9	1.455 (2)	C10—H10B	0.9299
N2—C10	1.281 (2)	O1—C11	1.338 (14)
C1—C2	1.365 (3)	O1—C14	1.373 (9)
C1—C6	1.372 (3)	C11—C12	1.396 (13)
C1—H1A	0.93	C12—C13	1.444 (18)
C2—C3	1.378 (3)	C12—H12A	0.93
C2—H2A	0.93	C13—C14	1.23 (3)
C3—C4	1.384 (2)	C13—H13A	0.93
C3—H3A	0.93	C14—H14A	0.93
C4—C5	1.378 (2)	O1'—C11'	1.320 (15)
C4—C7	1.502 (2)	O1'—C14'	1.349 (8)
C5—C6	1.379 (3)	C11'—C12'	1.347 (14)
C5—H5A	0.93	C12'—C13'	1.45 (2)
C6—H6A	0.93	C12'—H12B	0.93
C7—H7A	0.97	C13'—C14'	1.24 (3)
C7—H7B	0.97	C13'—H13B	0.93
C9—H9A	0.96	C14'—H14B	0.93
			
C8—S2—C7	102.08 (8)	H9A—C9—H9C	109.5
C8—N1—N2	115.50 (15)	H9B—C9—H9C	109.5
C8—N1—C9	123.00 (17)	N2—C10—C11'	128.2 (7)
N2—N1—C9	121.50 (16)	N2—C10—C11	114.4 (6)
C10—N2—N1	118.11 (17)	N2—C10—H10A	122.8
C2—C1—C6	119.30 (19)	C11'—C10—H10A	108.8
C2—C1—H1A	120.3	C11—C10—H10A	122.8
C6—C1—H1A	120.3	N2—C10—H10B	115.7
C1—C2—C3	120.25 (18)	C11'—C10—H10B	116.1
C1—C2—H2A	119.9	C11—C10—H10B	129.6
C3—C2—H2A	119.9	C11—O1—C14	108.6 (6)
C2—C3—C4	121.21 (18)	O1—C11—C12	106.8 (7)
C2—C3—H3A	119.4	O1—C11—C10	126.8 (9)
C4—C3—H3A	119.4	C12—C11—C10	126.3 (11)
C5—C4—C3	117.87 (18)	C11—C12—C13	104.3 (13)
C5—C4—C7	120.78 (17)	C11—C12—H12A	127.9
C3—C4—C7	121.35 (17)	C13—C12—H12A	127.9
C4—C5—C6	120.78 (18)	C14—C13—C12	109.3 (13)
C4—C5—H5A	119.6	C14—C13—H13A	125.4
C6—C5—H5A	119.6	C12—C13—H13A	125.4
C1—C6—C5	120.59 (19)	C13—C14—O1	111.0 (9)
C1—C6—H6A	119.7	C13—C14—H14A	124.5
C5—C6—H6A	119.7	O1—C14—H14A	124.5
C4—C7—S2	107.45 (12)	C11'—O1'—C14'	105.9 (6)
C4—C7—H7A	110.2	O1'—C11'—C10	120.0 (10)
S2—C7—H7A	110.2	O1'—C11'—C12'	111.8 (9)
C4—C7—H7B	110.2	C10—C11'—C12'	127.8 (13)
S2—C7—H7B	110.2	C11'—C12'—C13'	101.9 (15)
H7A—C7—H7B	108.5	C11'—C12'—H12B	129.1
N1—C8—S1	123.08 (14)	C13'—C12'—H12B	129.1
N1—C8—S2	113.05 (13)	C14'—C13'—C12'	108.9 (12)
S1—C8—S2	123.87 (11)	C14'—C13'—H13B	125.5
N1—C9—H9A	109.5	C12'—C13'—H13B	125.5
N1—C9—H9B	109.5	C13'—C14'—O1'	111.5 (9)
H9A—C9—H9B	109.5	C13'—C14'—H14B	124.3
N1—C9—H9C	109.5	O1'—C14'—H14B	124.3

### Hydrogen-bond geometry (Å, º)

Table 1. Cg is the centroid of the phenyl ring

**Table:**

*D*—H···*A*	*D*—H	H···*A*	*D*···*A*	*D*—H···*A*
C7—H7*B*···*Cg*^i^	0.97	2.88	3.560 (2)	128
C13—H13*A*···*Cg*^ii^	0.93	2.80	3.62 (2)	149

Symmetry codes: (i) −*x*−1, −*y*, −*z*+1; (ii) −*x*+1, −*y*, −*z*+2.
